# Primary Malignant Melanoma of the Esophagus Presenting as Chronic Cough

**DOI:** 10.14309/crj.0000000000000645

**Published:** 2021-08-17

**Authors:** Andrew L. Weskamp, Caleb B. Hudspath, Adam M. Tritsch

**Affiliations:** 1 Walter Reed National Military Medical Center, Bethesda, MA

## CASE REPORT

A 71-year-old man with a history of asthma and gastroesophageal reflux disease (GERD) presented to the emergency department (ED) with a chief complaint of new-onset right-sided abdominal tenderness attributed to chronic cough. Over the past 2 months, he had been treated with a proton pump inhibitor, leukotriene antagonist, and inhaled steroid without improvement. He also endorsed a 14-pound weight loss.

On presentation to the ED, laboratory test results were significant for a new elevation in liver-associated enzymes (aspartate transaminase 60 U/L and alanine transaminase 85 U/L), prompting right-upper quadrant ultrasound evaluation. The ultrasound revealed innumerable solid lesions within the liver, and a subsequent thoracic, abdominal, and pelvic computed tomography showed a 1.9-cm × 6.1-cm mass within the distal esophagus. The patient was admitted for expedited workup of suspected malignancy.

**Figure 1.**
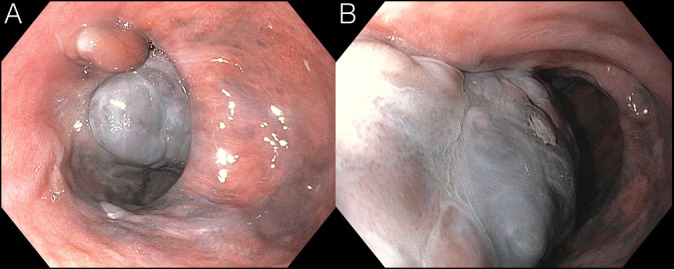
(A and B): Hyperpigmented mass in the distal esophagus from esophagogastroduodenoscopy.

**Figure 2.**
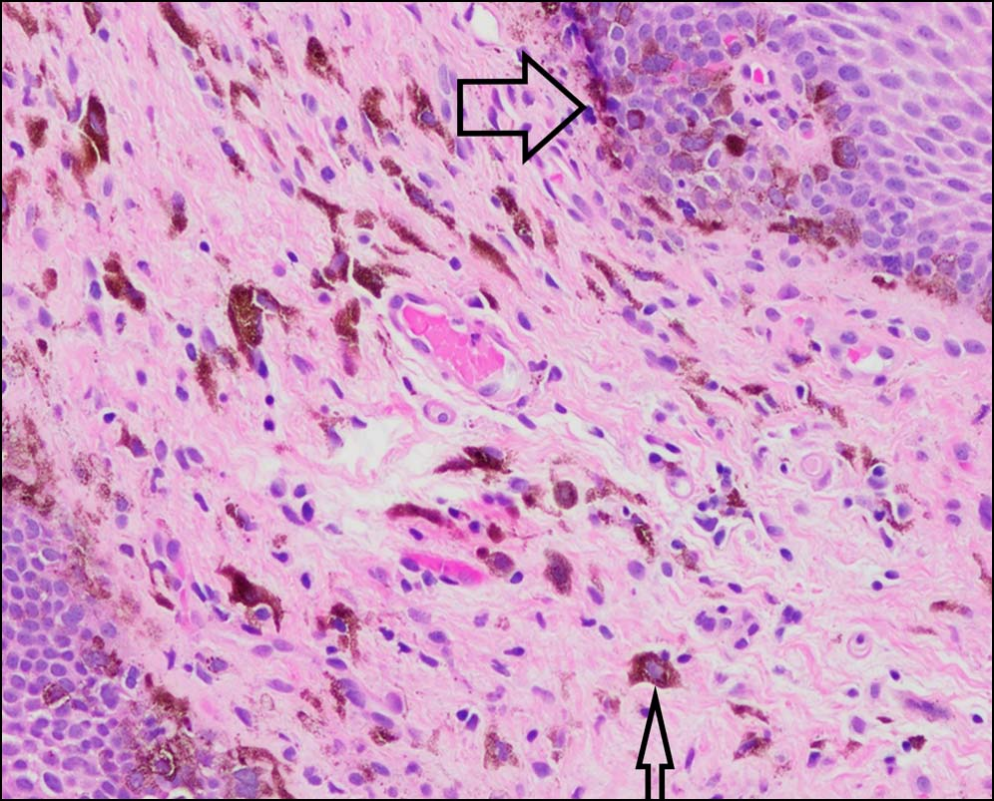
Biopsy, distal esophagus: basal layer proliferation of melanocytes, noted by their prominent nucleoli and adjacent melanin pigment (large arrow). Submucosal melanophages are visible, with notable pigment inclusion (small arrow).

An esophagogastroduodenoscopy (EGD) was performed which revealed a pigmented mass in the distal esophagus (Figure 1). The lesion had a vascular appearance, so biopsies were taken from the periphery of the mass and opposite wall; these demonstrated melanocytic proliferation but did not establish the diagnosis of malignant melanoma (Figure 2).

Interventional radiology-guided biopsies of the liver confirmed the diagnosis of metastatic malignant melanoma. The patient was diagnosed with T4bN3M1a BRAF-negative metastatic melanoma involving the esophagus, liver, mesentery, and lumbar spine. Detailed skin examination and formal ophthalmology evaluation did not reveal any primary lesions, establishing the diagnosis of primary malignant melanoma of the esophagus (PMME).

Given his metastatic disease burden, radical esophagectomy, the standard treatment for PMME, could not be performed. He was treated with 1 cycle of dual checkpoint inhibitor therapy with ipilimumab and nivolumab, a CTLA-4 and PD-1 inhibitor. The treatment course was complicated by autoimmune hepatitis. The patient declined additional chemotherapy, and he transitioned to comfort care.

PMME is an exceedingly rare disease with fewer than 400 cases reported worldwide. It comprises only 0.1%–0.02% of all esophageal malignancies.^1^ Moreover, it carries an extremely poor prognosis with a 5-year survival estimation from 4% to 37%.^2^ Treatment for PMME relies on early detection and esophageal resection, but new data suggest there may be survival benefits using checkpoint inhibitor therapy for metastatic disease.^2–4^ Patients' PMME typically presents with subacute or chronic dysphagia and epigastric pain,^5^ but this patient denied these symptoms and presented with the primary complaint of chronic cough.

## DISCLOSURES

Author contributions: AL Weskamp wrote the manuscript and is the article guarantor. CB Hudspath edited the manuscript. AM Tritsch edited the manuscript and approved the final manuscript.

Financial disclosure: None to report.

Previous presentation: This case was presented at the American College of Physicians Regional Conference, May 19, 2020; Virtual.

Informed consent could not be obtained from the family of the deceased patient despite several attempts. All identifying information has been removed from this case report to protect patient privacy.
